# Structural insights into Noonan/LEOPARD syndrome-related mutants of protein-tyrosine phosphatase SHP2 (*PTPN11*)

**DOI:** 10.1186/1472-6807-14-10

**Published:** 2014-03-14

**Authors:** Wei Qiu, Xiaonan Wang, Vladimir Romanov, Ashley Hutchinson, Andrés Lin, Maxim Ruzanov, Kevin P Battaile, Emil F Pai, Benjamin G Neel, Nickolay Y Chirgadze

**Affiliations:** 1 Princess Margaret Cancer Center, University Health Network, Toronto, Ontario, M5G 2C4, Canada; 2 Department of Medical Biophysics, University of Toronto, Toronto, Ontario, M5S 1A8, Canada; 3 Hauptman–Woodward Medical Research Institute, IMCA-CAT, Advanced Photon Source, Argonne National Laboratory, Argonne, Illinois 60439, USA; 4 Departments of Biochemistry, Molecular Genetics, and Medical Biophysics, University of Toronto, Toronto, Ontario, M5S 1A8, Canada; 5 Department of Pharmacology and Toxicology, University of Toronto, Toronto, Ontario, M5S 1A8, Canada

## Abstract

**Background:**

The ubiquitous non-receptor protein tyrosine phosphatase SHP2 (encoded by *PTPN11*) plays a key role in RAS/ERK signaling downstream of most, if not all growth factors, cytokines and integrins, although its major substrates remain controversial. Mutations in *PTPN11* lead to several distinct human diseases. Germ-line *PTPN11* mutations cause about 50% of Noonan Syndrome (NS), which is among the most common autosomal dominant disorders. LEOPARD Syndrome (LS) is an acronym for its major syndromic manifestations: multiple Lentigines, Electrocardiographic abnormalities, Ocular hypertelorism, Pulmonary stenosis, Abnormalities of genitalia, Retardation of growth, and sensorineural Deafness. Frequently, LS patients have hypertrophic cardiomyopathy, and they might also have an increased risk of neuroblastoma (NS) and acute myeloid leukemia (AML). Consistent with the distinct pathogenesis of NS and LS, different types of *PTPN11* mutations cause these disorders.

**Results:**

Although multiple studies have reported the biochemical and biological consequences of NS- and LS-associated *PTPN11* mutations, their structural consequences have not been analyzed fully. Here we report the crystal structures of WT SHP2 and five NS/LS-associated SHP2 mutants. These findings enable direct structural comparisons of the local conformational changes caused by each mutation.

**Conclusions:**

Our structural analysis agrees with, and provides additional mechanistic insight into, the previously reported catalytic properties of these mutants. The results of our research provide new information regarding the structure-function relationship of this medically important target, and should serve as a solid foundation for structure-based drug discovery programs.

## Background

**Figure 1:**
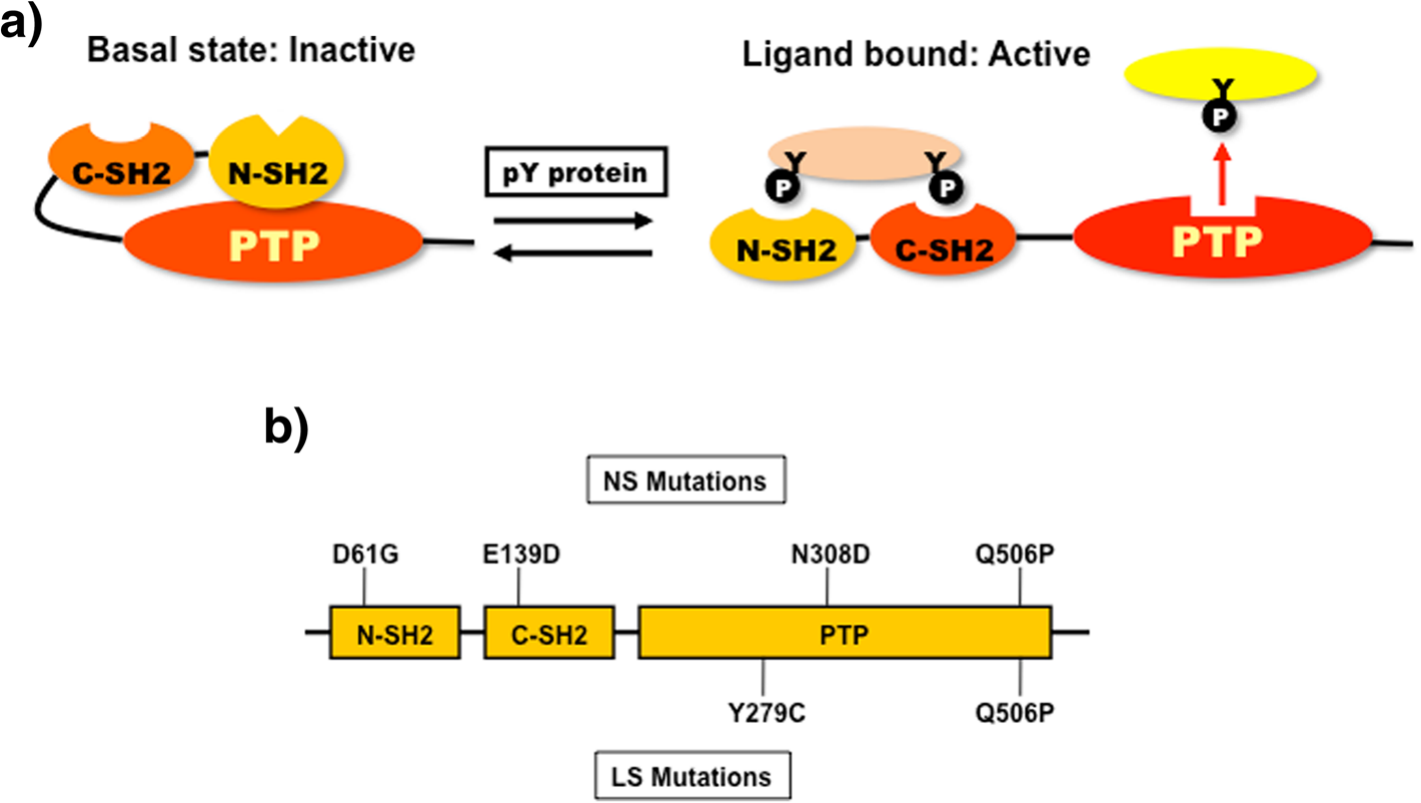
**SHP2 regulation and disease-associated mutants. a)** SHP2 is regulated via a “self-locking” mechanism: in the absence of pTyr- proteins (pY), SHP2 exists in a closed conformation with the N-SH2 domain bound to the PTP domain, blocking the catalytic site. Upon binding of the appropriate pTyr-proteins, the closed confirmation is disrupted, opening up SHP2 so that substrates can bind to the active site. **b)** Positions of human disease-associated mutants used in this study.

The ubiquitous non-receptor protein tyrosine phosphatase SHP2 (encoded by *PTPN11*) plays a key role in RAS/ERK signaling downstream of most, if not all growth factors, cytokines and integrins, although its major substrates remain controversial [1,2]. SHP2 contains two N-terminal SH2 domains, a catalytic (PTP) domain, a C-terminal tail with two tyrosine phosphorylation sites and a proline-rich domain [2-5], and is regulated by an elegant molecular switch mechanism that couples appropriate cellular localization to catalytic activation [3,5]. In the absence of a tyrosine-phosphorylated binding partner for its SH2 domains (basal state), SHP2 assumes a “closed” conformation wherein the N-terminal SH2 (N-SH2) domain is wedged into the PTP domain, blocking substrate access (Figure 1a). Upon agonist stimulation, recruitment of the N-SH2 domain to specific phosphotyrosyl (pTyr-) peptides disrupts this self-locking conformation, freeing the PTP domain for catalysis [3,5,6].

Mutations in *PTPN11* cause several human diseases. Germ-line *PTPN11* mutations cause ~50% of Noonan Syndrome (NS), which is among the most common autosomal dominant disorders [7,8]. Gain-of-function mutations in other RAS-RAF-MEK-ERK pathway members, including *SOS1*[9,10]*, KRAS*[11]*, NRAS*[12], *SHOC2*[13], and *RAF1*[14,15], are responsible for most remaining NS cases. With an estimated incidence of 1/2,000 live births [16], NS is characterized by facial dysmorphism, proportional short stature, cardiac anomalies, and various less penetrant phenotypes, such as webbed neck, deafness, and motor delay. Many (20-50%) NS patients develop some type of myeloproliferative disorder (MPD), which is typically mild and self-limited [17]. Rare NS patients progress to Juvenile Myelomonocytic Leukemia (JMML), which is fatal if not treated by bone marrow transplantation, somatic *PTPN11* mutations are the single most common cause of sporadic JMML [7,18-20]. LEOPARD Syndrome (LS), a much less common autosomal dominant disorder, is almost always caused by *PTPN11* mutations, and is related to, but distinguishable from, NS [7,16,21]. LEOPARD is an acronym for its major syndromic manifestations: multiple Lentigines, Electrocardiographic abnormalities, Ocular hypertelorism, Pulmonary stenosis, Abnormalities of genitalia, Retardation of growth, and Deafness [22]. These patients often have hypertrophic cardiomyopathy (HCM), and might also have an increased risk of neuroblastoma (NS) and acute myeloid leukemia (AML) [23,24]. Knock-in mouse models have been generated for NS and LS alleles of *Ptpn11* and generally reproduce the phenotypes seen in the cognate human syndromes [25-27].

Consistent with the distinct pathogenesis of NS and LS, different types of *PTPN11* mutations cause these disorders. Most NS-associated *PTPN11* mutations alter residues that reside at the interface between the N-SH2 and PTP domains [16], resulting in elevated enzymatic activity and enhanced RAS/ERK activation [27-31]. These data suggest that NS mutations disrupt the intramolecular interaction between the N-SH2 and PTP domains, shifting the equilibrium between the closed and open conformations and lowering the activation threshold for SHP2. By contrast, LS mutations typically affect PTP domain residues, result in markedly decreased catalytic activity, and lower RAS/ERK activation in transient transfection assays [31-33]. Studies of the LS Y279C mouse model also indicate that LS mutants may have dominant negative effects in at least some tissues *in vivo*[25]. Whereas NS phenotypes arise from enhanced MEK/ERK activation and can be prevented or reversed by MEK inhibition [34-36], LS-associated HCM is caused by enhanced PI3K/AKT/mTORC1 activity and can be reversed by rapamycin [25].

Although multiple studies have reported the biochemical and biological consequences of NS- and LS-associated *PTPN11* mutations, their structural consequences have not been analyzed. Here, we report the X-ray structures of five NS/LS SHP2 mutants and discuss how these mutations affect the interaction between different SHP2 domains and its catalytic activity.

## Methods

### Cloning

A wild type (WT) SHP2 expression construct 1–539 (comprising the N + C SH2 and PTP domains) was PCR-amplified from *PTPN11* cDNA [37] with a set of custom-designed primers (see Additional file 1: Description S1). The resultant PCR fragment was cloned into a modified version of the plasmid pET28b (Novagen) that generates a fusion protein with an N-terminal hexahistidine tag. The SHP2 catalytic domain expression construct (a.a. 221–524) was cloned into pGEX4T, which introduces a GST-tag at its N-terminus. Mutations were introduced into these expression constructs by site directed-mutagenesis with specifically designed primers bearing one substitution each (see Additional file 1: Description S1). Pfu Ultra II high fidelity DNA polymerase (Stratagene) was used for PCR, with an extension temperature of 68°C over 10 minutes. To remove any traces of the original cDNA, all reactions were subjected to digestion with *DpnI* (New England Biolabs) for 1 hour at 37°C. Reaction mixtures were transformed into DH5α cells, and the genetic content of all constructs was verified by Sanger sequencing.

### Protein expression & purification

Vectors encoding full length versions of SHP2 mutants were transferred into *E. coli* BL21(DE3). Cells were grown in Terrific Broth containing kanamycin (50 mg/l) in 1 L Tunair flasks at 37°C to an OD_600_ of 3–5, after which the temperature was lowered to 16°C, and isopropyl-1-thio-β-D-galactopyranoside (IPTG) was added to 0.2 mM. Expression was allowed to proceed overnight, then cells were harvested by centrifugation, flash-frozen in liquid nitrogen, and stored at −80°C. Due to the low level of expression of the Q506P construct, cells expressing this mutant were washed using the osmotic shock technique [38] prior to freezing. Unless stated otherwise, all purification procedures were carried out at 4°C. Cells were thawed on ice and resuspended in Binding Buffer (see Additional file 2: Table S1 for detailed buffer components), supplemented with phenylmethylsulfonylfluoride and benzamidine. After disruption by sonication and centrifugation at 60,000 g for 40 min, cell-free extracts were passed through a DE-52 column (2.6 × 7 cm) that had been pre-equilibrated with the same buffer, and loaded by gravity flow onto a Ni-nitrilotriacetic acid (NTA) column (Qiagen, Germantown, MD). The latter column was washed with 20–25 volumes of Wash Buffer A, followed by 20–25 volumes of Wash Buffer B and finally with Elution Buffer. N308D and E139D eluted in Elution Buffer, whereas the other three mutants eluted with the Wash buffers. For the latter proteins, the wash fractions were diluted 15-fold and reloaded on fresh Ni-NTA columns. After washing with 10 column volumes of Binding Buffer, N308D and E139D proteins were eluted in elution buffer. These samples were concentrated using a VIVASpin unit (Sartorius NA, Edgewood, NY), and loaded onto a 2.6 × 60 cm Superdex 200 column (GE Healthcare), equilibrated with Gel Filtration buffer. Elution was performed at a flow rate of 3 ml/min at 8°C, with the SHP2 proteins behaving as apparent monomers. Final protein samples were concentrated to 20–40 mg/ml, divided into 1.5 mg aliquots, flash-frozen and stored at −80°C.

SHP2 catalytic domain mutants were transformed into *Escherichia coli* strain BL21(DE3). A 25 ml aliquot of an overnight culture from a single colony was added to 500 ml of LB/ampicillin (50 μg/ml) and grown at 37°C to A_600_ = 0.8. IPTG was added to a final concentration of 0.1 mM, and the bacteria were maintained for 16 h at 25°C with shaking, then centrifuged at 6,000 × g for 10 min. at 4°C. Pellets were resuspended in 12.5 ml of a buffer containing 50 mM Tris–HCl, pH 7.5, 150 mM NaCl, 5 mM MgCl2, 1% Triton X-100, 10% glycerol, 5 mM dithiothreitol, 2 μg/ml aprotinin, 10 μg/ml leupeptin, 1 μg/ml antipain, 1 μg/ml pepstatin A, 0.5 mg/ml lysozyme and 1 mg/mL DNase I. Suspensions were incubated on ice for 30 minutes, and then sonicated for 10 seconds on ice. Lysates were centrifuged at 14,000 × g for 30 min. at 4°C, and supernatants were transferred to a fresh 15-ml polypropylene tube containing 0.5 ml of glutathione-Sepharose 4B (GE Healthcare Life Sciences). This suspension was rotated end-over-end overnight at 4°C, and then centrifuged at 1000 × g for 1 min. at 4°C. The supernatants were discarded, and the beads were washed 3 times for 5 min. each at 4°C with 10 ml of wash buffer (25 mM Tris–HCl, pH 7.5, 150 mM NaCl, 5 mM MgCl2, 1% Triton X-100, 10% glycerol, 5 mM dithiothreitol), and then once with PTP assay buffer (25 mM Hepes, pH 7.5, 100 mM NaCl, 2 mM EDTA and 5 mM dithiothreitol). Bound GST fusion proteins were resuspended 1:1 in PTP assay buffer. A 20 uL aliquot of slurry for each mutant was separated on a 10% SDS-polyacrylamide gel, together with different amounts of BSA. The gel was washed in water for 10 minutes, and stained with Colloidal Coomassie Blue for 1 hour at room temperature. Bands were quantified using a LI-COR Odyssey.

### PTP assays

To determine kinetic parameters, fixed amounts of purified GST-WT or -mutant SHP2 catalytic domains (1.6 pmol of WT and N308D, 115pmol of Y279C and 16.3 pmol of Q506P) were incubated with variable concentrations of substrate peptide (R-R-L-I-E-D-A-E-pY-A-A-R-G, Millipore #12-217; Kit #12-217) in PTP assay buffer in a total volume of 50 uL. Reactions were carried out for 10 minutes at 25°C, and phosphate release was quantified by adding Malachite Green (Millipore #17-125) to the supernatants, measuring absorbance at 620 nm, and comparing values to a standard curve generated with varying amounts of KH_2_PO_4_. All reactions fell within the linear range. Phosphatase activity is expressed in pmol Pi released/min/pmol enzyme.

### Crystallization

Mutant SHP2 proteins were crystallized under conditions similar to those reported previously [3]. In order to obtain the best diffracting crystals, 0.1 M LiCl was added to the literature crystallization conditions for D61G, 5% glycerol for N308D, and 10% glycerol and 0.3 M cycohexyl-methyl-β-D-maltoside for Q506P. The other two mutant proteins and the WT protein were crystallized under literature conditions with optimized precipitant concentrations. Crystals appeared overnight, and reached their full size of about 300 × 300 × 30 microns in one week at room temperature. The stacked plate crystals were separated and flash frozen in liquid nitrogen, using paratone-N oil (Hampton Research Inc.) as a cryo-protectant.

### Data collection

Data were collected at 100 K with a wavelength of 1.0 Å on the Industrial Macromolecular Crystallography Association (IMCA-CAT) beam line at the Advanced Photon Source (Argonne National Laboratory, IL USA). The data were indexed, integrated, and scaled with *XDS* and *XSCALE*[39].

### Structure determination

The first mutant structure of N308D was determined by molecular replacement, using the previously solved structure of SHP2 (PDB access code 2SHP) [3] as the search model. The wild type SHP2 and D61G, E139D, Y279C and Q506P mutant structures were determined by molecular replacement, using the structure of the N308D SHP2 mutant as a search model. Following the initial rigid body refinement, interactive cycles of model building and refinement were performed by using *COOT*[40] and *Buster-TNT*[41] software. Special attention was paid to the mutation sites, which were initially replaced with alanine to reduce model bias and later positioned based on the *2mF*_*o*_*-DF*_*c*_ and difference Fourier electron density maps after a few rounds of refinement to confirm that those amino acids had, indeed, been mutated. Data collection and refinement statistics are shown in Table 1. The atomic coordinates have been deposited in the RCSB Protein Data Bank under accession numbers 4NXD, 4H10, 4NWG, 4GWF, 4NWF, and 4H34. All figures were produced using *PyMOL* (http://www.pymol.org).

**Table 1:** Summary of crystallographic data and refinement statistics

**Parameters**	**Wild type**	**D61G**	**E139D**	**Y279C**	**N308D**	**Q506P**
*Data collection:*						
Resolution, (Å)	2.75	2.20	2.45	2.10	2.10	2.70
Outermost resolution shell, (Å)	(2.85-2.75)	(2.30-2.20)	(2.55-2.45)	(2.20-2.10)	(2.20-2.10)	(2.80-2.70)
Space group	P2_1_	P2_1_2_1_2	P2_1_2_1_2_1_	P2_1_	P2_1_2_1_2_1_	P2_1_2_1_2
Unit cell parameters						
*a,* (Å)	55.7	55.0	56.3	55.7	55.9	54.8
*b,* (Å)	211.7	220.3	212.4	212.0	211.2	202.4
*c,* (Å)	91.2	41.7	92.2	46.0	91.6	44.5
β, (°)	89.97			96.6		
Molecules per asymmetric unit	4	1	2	2	2	1
Unique reflections	53,849	26,689	41,625	61,515	64,401	14,342
Multiplicity	3.1 (3.4)	6.3 (6.1)	6.5 (6.3)	3.5 (3.5)	7.0 (7.2)	6.5 (6.3)
Average I/σ (I)	5.5 (2.2)	11.1 (2.7)	9.2 (2.3)	6.9 (1.9)	11.0 (3.2)	12.0 (2.8)
*R*_*merge*_*,* (%)	18.9 (46.0)	10.6 (52.0)	11.4 (57.1)	10.4 (49.7)	9.4 (43.0)	13.9 (56.0)
Completeness, (%)	95.9 (98.5)	99.3 (96.2)	99.7 (98.1)	99.9 (99.6)	99.9 (100)	99.9 (100)
*Refinement and structure statistics*						
*R*_*work*_*,* (%)	25.6	21.1	21.3	21.1	24.2	22.7
*R*_*free*_*,* (%)	28.5	23.3	25.4	24.3	28.7	24.2
RMSD from ideal geometry						
Bond lengths, (Å)	0.007	0.007	0.010	0.008	0.009	0.007
Bond angles, (°)	0.91	0.99	1.24	1.00	1.10	0.91
Numbers of atoms						
Protein (non-hydrogen)	15,560	4,021	8,214	8,013	8,100	3,951
Water oxygen atoms	784	134	365	261	679	54
Ligand’s atoms		20		90		36
PDB ID	4NXD	4H10	4NWG	4GWF	4NWF	4H34

*R*_*merge*_ = ∑ _*hkl*_|*I* − 〈*I*〉|/∑_*hkl*_*I*, where *I* is the intensity of the individual reflections.

*R*_*work*_ = ∑ |*F*_*obs*_ − *F*_*cal*_|/∑|*F*_*obs*_, where *F*_obs_ and *F*_calc_ are the observed and the calculated structure factors, respectively.

*R*_*free*_ was calculated using 5% of total reflections randomly chosen and excluded from the crystallographic refinement.

## Results and discussion

We determined the crystal structures of WT SHP2 (residues 1–539), as well as five mutants (D61G, E139D, Y279C, N308D, and Q506P), chosen to represent the spectrum of disease-associated *PTPN11* mutations. Mutants D61G, E139D, and N308D are found in NS, Y279C is a canonical LS mutation [16,21,42], and Q506P has been reported in both disorders [16], although it is unclear whether this reflects misdiagnosis or true bi-potentiality of this allele. The D61G mutation affects the N-SH2 domain, E139D lies within the C-SH2 domain and the other three mutations alter the PTP domain (Figure 1b). The enzymatic properties of the full-length versions of these mutants (including the C-terminal tail, which is missing in our crystallization constructs) were characterized previously by our group [29,33] (Additional file 3: Figure S1), and range from strongly activated (D61G), to mildly activated (N308D), to catalytically impaired (Y279C). Q506P shows altered specificity for some substrates [29].

**Figure 2:**
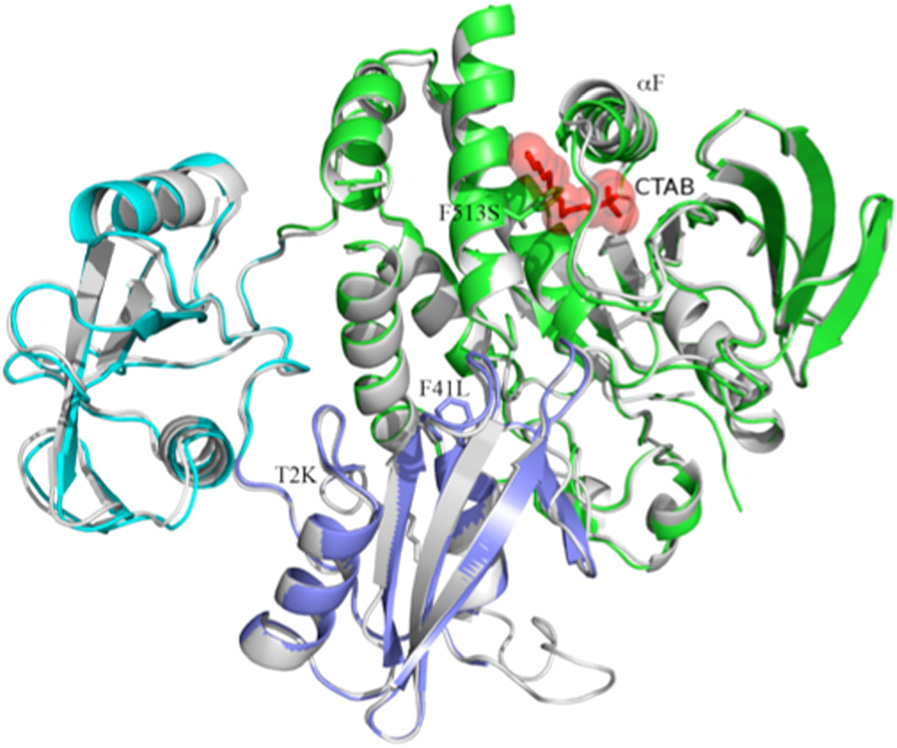
**Comparison of “true” WT SHP2 structure (gray) with previously determined “WT” structure (PDB accession code: 2SHP).** Note that the earlier structure has three mutations (T2K, F41L and F513S, presented in red sticks) and also contains a molecule of the detergent CTAB, (presented as red sticks and transparent sphere). The F513S mutation creates a cavity that binds the detergent molecule, which alters the orientation of the αF helix.

The SHP2 structure published by Hof *et al.* (PDB accession code: 2SHP; hereafter termed “2SHP”) has three mutations (T2K, F41L and F513S) and is in complex with a detergent molecule (CTAB). We corrected these mutations, and crystallized WT SHP2 under detergent-free conditions. In our WT structure, the SH2 domains and the PTP domain assume a “closed” conformation with the N-SH2 domain locked into the PTP catalytic site, similar to the 2SHP structure (Figure 2). Superimposition of our *bona fide* WT structure with the earlier “WT” SHP2 structure revealed an overall root mean square deviation (*rmsd*) of 0.59 Å over 487 aligned residues. The N-SH2 domain had a smaller *rmsd* of 0.35 Å, whereas the *rmsd* for the C-SH2 was larger (0.74 Å), implying that the C-SH2 domain might have a higher degree of flexibility than the other two domains in the closed conformation. The three mutations and the CTAB binding site in the 2SHP structure are distant from the C-SH2 domain, so we do not think it likely that they affect C-SH2 domain flexibility directly. In the PTP domain, the major deviations between the two structures were seen in residues 425–436 of the αF helix (average *rmsd* =1.33 Å), corresponding to the CTAB binding site in the 2SHP structure (Figure 2). Although the overall structural difference between WT and 2SHP was small, we used our WT structure as the reference for comparison with the mutant structures to exclude any potential structural changes induced by the three mutations and the detergent molecule (CTAB) in 2SHP.

### D61G

**Figure 3:**
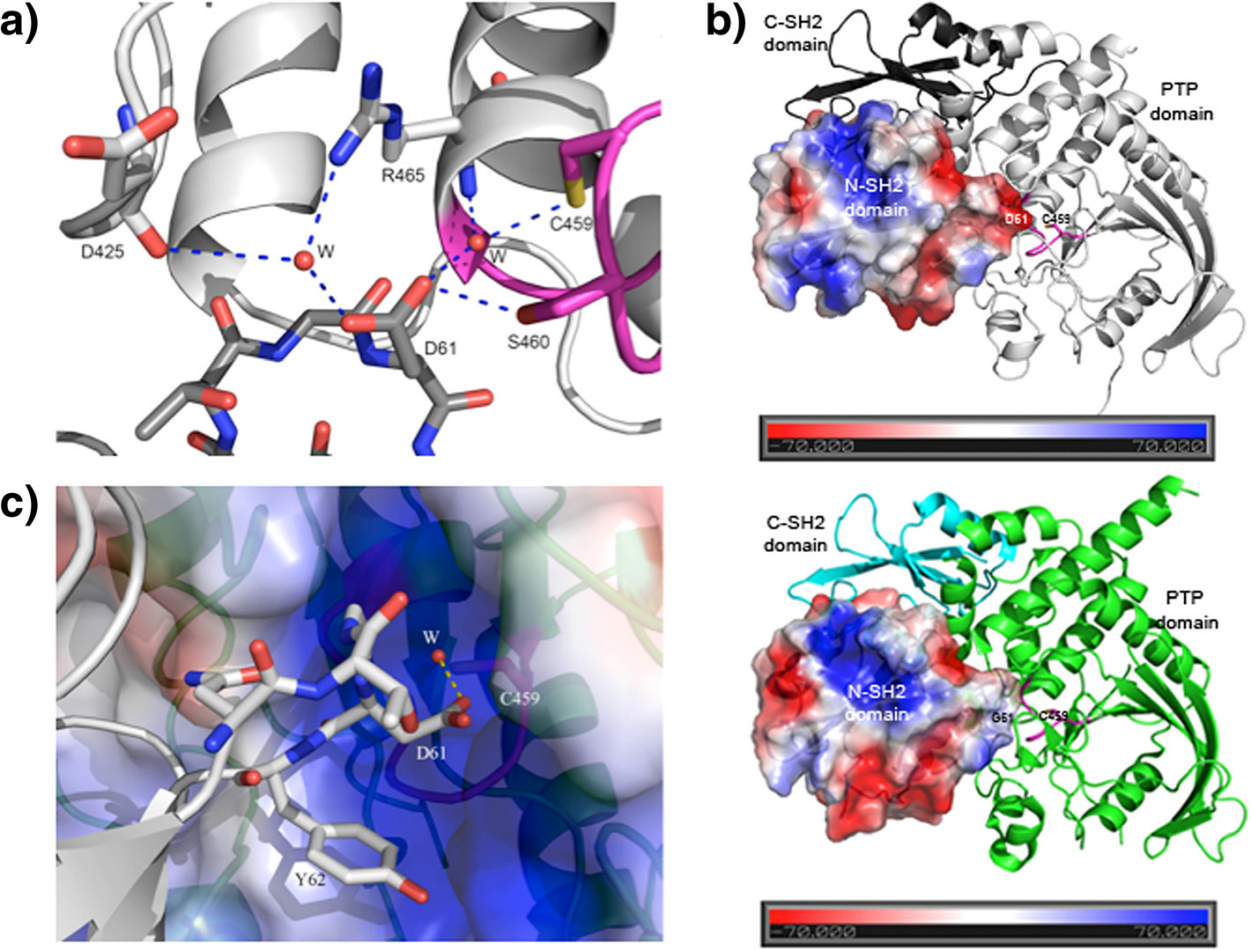
**Crystal structure of the D61G mutant. a)** In WT SHP2, the side chain of Asp61 (in the N-SH2 domain) forms a direct hydrogen bond with Ser460, a water-mediated hydrogen bond with the catalytic cysteinyl residue Cys459 and two water-mediated hydrogen bonds with Arg465 in the PTP domain. **b)** The D61G mutation alters the electrostatic surface charge on the N-SH2 domain along its interaction interface with the PTP domain catalytic pocket. The N-SH2 domain is rendered in electrostatic surface representation, the C-SH2 domain is colored dark grey (WT structure; top panel) or cyan (D61G mutant structure; bottom panel), and the PTP domain is shown in grey (WT) or green (D61G). The catalytic P-loop (458–464) is shown in magenta. **c)** The N-SH2/PTP domain interface near the catalytic site. The PTP domain is rendered in electrostatic surface presentation, and shows a mostly positively charged catalytic site opposite to Asp61. A conserved water molecule mediates a hydrogen bond between Asp61 and Cys459.

In the WT structure, Asp61 was located on the surface of the N-SH2 domain. The side chain of Asp61 formed hydrogen bonds with Ser460 from the catalytic P-loop (residues 458–464), a water-mediated hydrogen bond with the catalytic cysteinyl residue, Cys459, two water-mediated hydrogen bonds with Arg465, and another water-mediated hydrogen bond with Asp425 (Figure 3a). Consequently, Asp61 plays an important role in the N-SH2 and PTP domain interaction. In the D61G mutant structure, these hydrogen bonds were abolished. The change from aspartate to glycine also altered the surface charge from very negative to neutral (Figure 3b). Opposite D61G on the interface surface, the PTP domain presents a predominantly positively charged pocket (Figure 3c). Thus, the D61G mutation greatly loosened the interactions between N-SH2 and PTP domains. These data are consistent with previous publications that observed increased basal activity for this mutant against artificial (e.g., pNPP) and pTyr-peptide substrates [29,33].

### E139D

**Figure 4:**
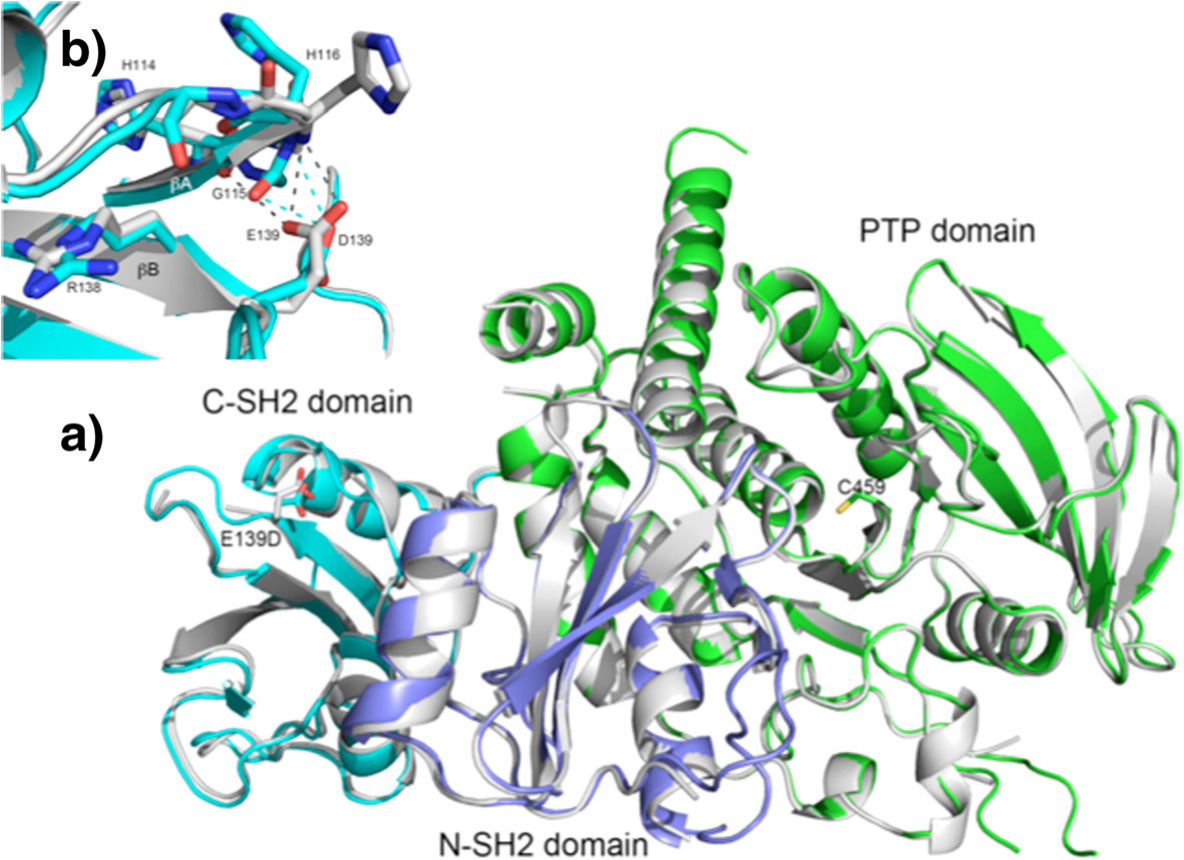
**Crystal structure of the E139D mutant. a)** Crystal structure of the E139D mutant does not display any obvious conformational changes in the C-SH2 (shown in cyan) or PTP domains (shown in green), superimposed on the WT SHP2 structure (colored in grey). Note that the mutant residue E139D is far away from the catalytic cysteine, Cys459 (~40 Å). The N-SH2 domain is shown in blue. **b)** In the E139D structure, the side chain of Asp139 forms only two hydrogen bonds with the main chain atoms of G115 and His116 (cyan dash lines), whereas in the WT structure, Glu139 forms three hydrogen bonds with His114 and His116 (grey dash lines). The mutation could loosen the connection between the βA and βB strand in C-SH2 domain, thereby exposing the side chain of Arg138, a key residue for pTyr-peptide binding.

Residue Glu139 was located on the surface of the C-SH2 domain. The overall crystal structure of the E139D mutant was very similar to that of WT SHP2, with an *rmsd* of 0.4 Å (Figure 4a). Glu139 was about 40 Å away from the catalytic site, with the N-SH2 domain interposed between these domains. E139 is, however, located in the vicinity of the phosphate group of the pTyr peptide-binding site of the C-SH2 domain, and the E139D mutation stabilizes the conformation of the 139–147 loop that plays an essential role in pTyr peptide-binding. The mutant structure has well defined electron density for this loop, whereas this loop is disordered in the WT structure. Compared with WT SHP2, there also were some local structural rearrangements in the E139D mutant, with the most noticeable difference being a conformational change of His116 (Figure 4b). The E139D mutant shows a small increase in basal activity, but when it is assayed with a pTyr peptide (pTyr1172 from IRS-1) that can bind both SH2 domains and be dephosphorylated by the catalytic domain, the activity of this mutant is more than 5 times higher than that of WT SHP2 [29]. Although the side chain charge remained unchanged when glutamic acid was changed to aspartic acid, the size of the side chain was reduced. This change could alter the surface of the adjacent residue, Arg138, a key residue for pTyr binding [29]. As shown in Figure 4b, aspartic acid 139 (located in the βB strand) contributes less hydrogen bonding to residues His114-His116 of the βA strand than does glutamic acid. This subtle change might loosen the connection between the βA and βB strands, helping to expose the side chain of Arg138, and thus enhancing the affinity for pTyr-peptide binding. The binding of pTyr -peptides to the C-SH2 domain also could affect the interaction between the N-SH2 and C-SH2 that is critical for enzymatic activation [43]. The mutant also could facilitate the binding of the C-SH2 domain to certain physiologically important binding partners; e.g., IRS-1, in which pTyr-1222 binds to the C-SH2 domain, while pTyr-1172 binds to the N-SH2 domain. The E139D mutation could indirectly increase the binding affinity of the N-SH2 domain for SHP2 substrates (for the reasons discussed above), and therefore increase catalytic activity. In concert, these effects likely explain why E139D is activated by pTyr-peptide binding more than WT SHP2.

### Y279C

**Figure 5:**
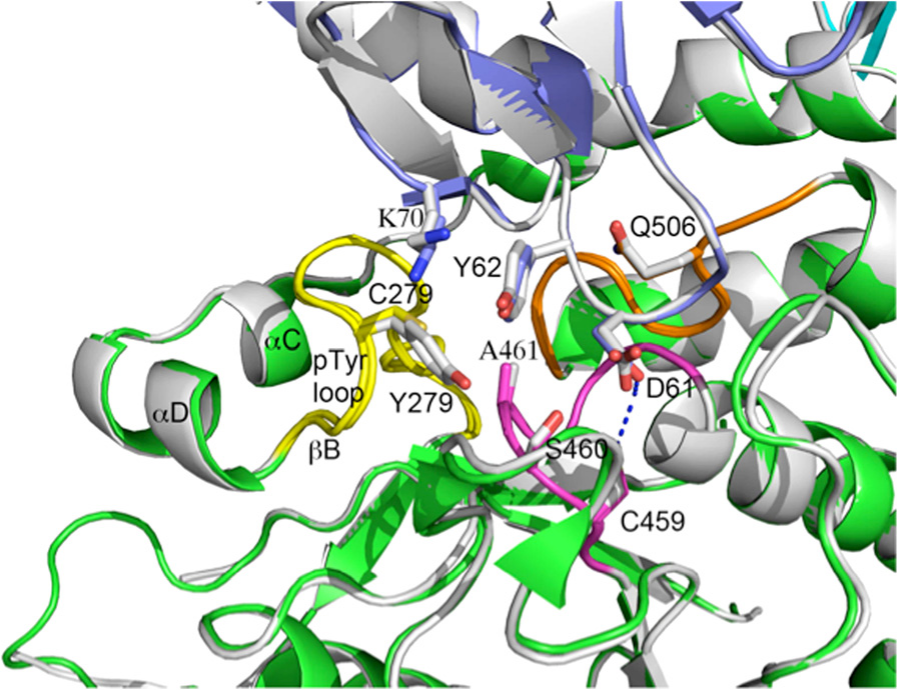
**Superimposition of the Y279C mutant and WT SHP2 (grey) structures.** In WT SHP2, Tyr279 lies in proximity to Cys459 and also interacts with Tyr62 of the N-SH2 domain (shown in blue and dark grey, respectively). Mutation from Tyr to Cys decreases these interactions. The P-loop (458–464) and pTyr loop (277–288) are highlighted in magenta and yellow, respectively.

Y279C is a catalytically impaired mutant associated with LS [16,21,42]. In the crystal structure of this mutant, Tyr279 was located in the long pTyr loop (residues 277–288) with two small α-helices, αC (residues 265–269) and αD (residues 271–276), at its upstream end and one small β-strand, βB (residues 289–292), at its downstream end. In general, because the intervening region (residues 262–288) lacks a structurally stable long α-helix or β-strand, it is likely to be flexible. It also contained a large number of positively charged side chains pointing toward the surface of the PTP domain that might interact with solvent molecules or other binding partners and thus increase the mobility of the pTyr loop. In the WT structure, the side chain of Tyr279 makes *van der Waals* contacts with Ser460 and Ala461 of the catalytic P-loop, as well as with Tyr62 and Lys70 from the N-SH2 domain (Figure 5). The –OH group of Tyr62 interacts with the π-electrons of the Tyr279’s aromatic ring. Together with Q506 in the Q-loop (residues 501–507), Tyr279 is believed to play a key role in binding the tyrosine side chains of substrate proteins/peptides during catalysis. In the PTP1B structure [44], Tyr46 is the residue equivalent to Tyr279 in the SHP2 structure [3,45]. The stacking interaction of three residues, Tyr46, the substrate pTyr, and Phe182 from the “WPDF” loop, help to properly position the substrate for catalysis. In the Y279C structure, the interactions of Tyr279 with the P-loop and the N-SH2 domain were disrupted, due to the significantly shorter side chain of cysteine compared with that of tyrosine. SHP2 has a WPDH loop that corresponds to WPDF in PTP1B. The Y279C mutation would have less stacking interactions with Tyr279, a bound pTyr substrate, and His426 (the equivalent of Phe182 in PTP1B). The mutation also distorts the pTyr substrate/SHP2 interaction, and thus would be expected to disrupt catalysis significantly. At the same time, the Y279C mutation results in loss of the Tyr279/Tyr62 interaction, diminishing the strength of intramolecular binding between the PTP and N-SH2 domains. This would be expected to facilitate the “open” conformation, and can explain the enhanced interaction of this mutant with binding partners (e.g., GAB1) observed previously [33,45,46]. While this manuscript was in preparation, Yu *et al*. [46] reported a very similar model of Y279C (PDB accession code: 4DGX), as well as WT SHP2 (PDB accession code: 4DGP). The Cα carbon atom comparison for these structures revealed RMSD of 0.42 and 0.47 Å for the corresponding mutant and wild-type crystal structure pairs correspondingly. Both published crystal structures belong to the P2_1_2_1_2 space group, which is different from the space group (P2_1_) for our Y279C mutant and WT structures. Importantly, the residues surrounding the mutant residue are in a similar conformation. Our WT structure has two disordered regions (perhaps due to their flexibility) lacking electron density (88–95 and 156–164). From both Y279C structures, we can see that replacing tyrosine with cysteine at position 279 does not block the accessibility to the PTP catalytic site; instead, it could facilitate local conformational changes that lead to the release of the N-SH2 domain, and thereby open the conformation of the substrate-binding site.

To test the predictions of these structural studies, we assayed the enzymatic activity of isolated SHP2 catalytic domains (present as part of GST-fusion proteins). Compared with WT GST-SHP2, the Y279C mutant had ~80 times lower k_cat_ and 7 times higher K_m_ towards a pY peptide (Figure 5, Table 2). These results are quite similar to those were reported by Yu *et al.*[46]. Yu *et al.* also provided hydrogen/deuterium exchange mass spectrometry experiments and molecular dynamics simulations showing that the N-SH2 and PTP domain interaction was decreased in the Y279C mutant. Moreover, they found that the Y279C mutant displayed higher affinity for, and was preferentially activated by, a non-hydrolyzable N-SH2 ligand.

**Table 2:** Catalytic activities of the indicated SHP2 catalytic domain (221–524) GST fusion proteins were measured using the Malachite Green assay in the presence of different concentrations of PTP-1B peptide R-R-L-I-E-D-A-E-pY-A-A-R-G. K_m_ and k_cat_ calculated by: 1/V = (K_m_/V_max_) / [pY] + 1/ V_max_

	**k**_**cat **_**(s**^**−1**^**)**	**K**_**m **_**(mM)**	**k**_**cat **_**/ K**_**m **_**(s**^**−1**^ **mM**^**−1**^**)**
WT	9.26	1.31	7.07
N308D	12.82	1.92	6.68
Y279C	0.12	9.14	0.01
Q506P	0.61	2.83	0.22

### N308D

**Figure 6:**
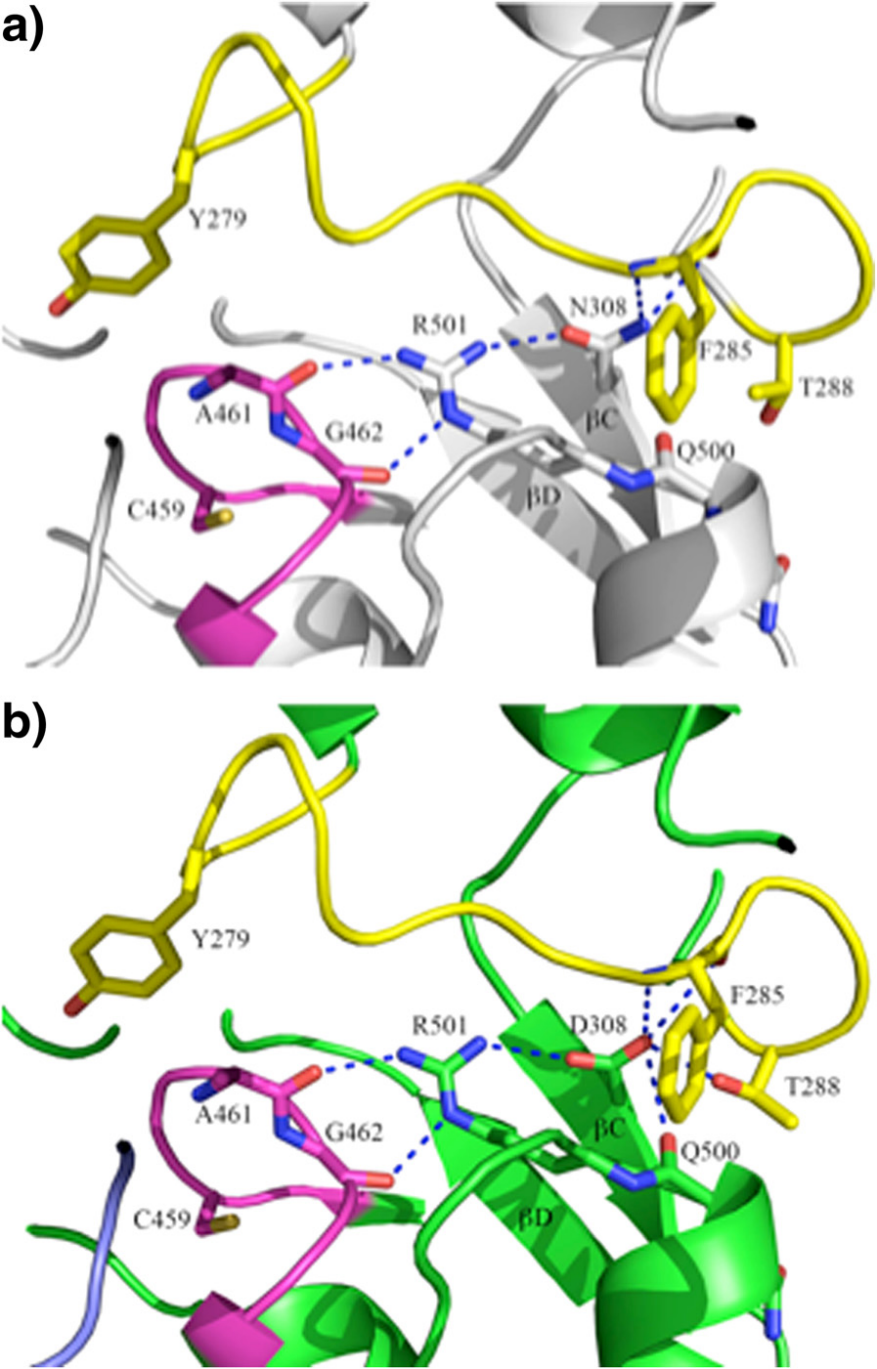
**N308D mutation alters the local hydrogen bond network. a)** In WT SHP2, the side chain of Asn308 forms two hydrogen bonds with Phe285 in the pTyr-loop (colored in yellow). It also forms a hydrogen bond with the conserved Arg501 which, in turn, makes two direct hydrogen bonds with the main chains of Ala461 and Gly462 in the catalytic P-loop (colored in magenta); **b)** In the N308D mutant, besides the aforementioned hydrogen bonds, Asp308 forms two additional hydrogen bonds with surrounding residues, most notably, a strong hydrogen bond with Thr288 (2.5 Å).

Residue 308 was located in the βC strand of the PTP domain, and was not involved in direct interactions with the N-SH2 domain. However, the Oδ1 atom from the side chain of Asn308 formed a strong hydrogen bond with the side chain of the conserved Arg501. Arg 501 also made direct hydrogen bonds with the main chains of the P-loop residues Ala461 and Gly462 (Figure 6a). The Nδ2 atom of Asn308 formed two hydrogen bonds with the main chain of Phe285 from the pTyr-loop. In the N308D mutant, the charge of the side chain changes from neutral to negative, whereas the side chain polarity changes from polar to acidic polar. Compared with the WT Asn residue, Asp308 formed more hydrogen bonds with pTyr-loop residues, and an especially strong one (2.5 Å) with Thr288 (Figure 6b). Consequently, this mutation could make the pTyr- and P-loops less flexible, locking the enzyme in a more favorable position for catalysis. The greater rigidity of the pTyr and P loops makes it more difficult for the N-SH2 domain to close back on the PTP domain once it opens. Since the open and closed forms are in equilibrium, this could mean that the ability (*i.e.,* rate constant) for closing back is significantly diminished, hence favoring the open form, leading to the increased activity (basally and in response to pTyr peptide) of the N308D mutant as a full-length enzyme. The residue Asn308 is a hot spot for NS mutations, with N308D accounting for 25% of NS cases. Previous studies showed that this mutant (as a full length protein) has a 3-fold higher basal activity than WT [29]. Our PTP assay showed that catalytic domain of this mutation had slightly higher k_cat_ and K_m_ values when compared with the WT PTP domain (Figure 1, Table 2).

### Q506P

**Figure 7:**
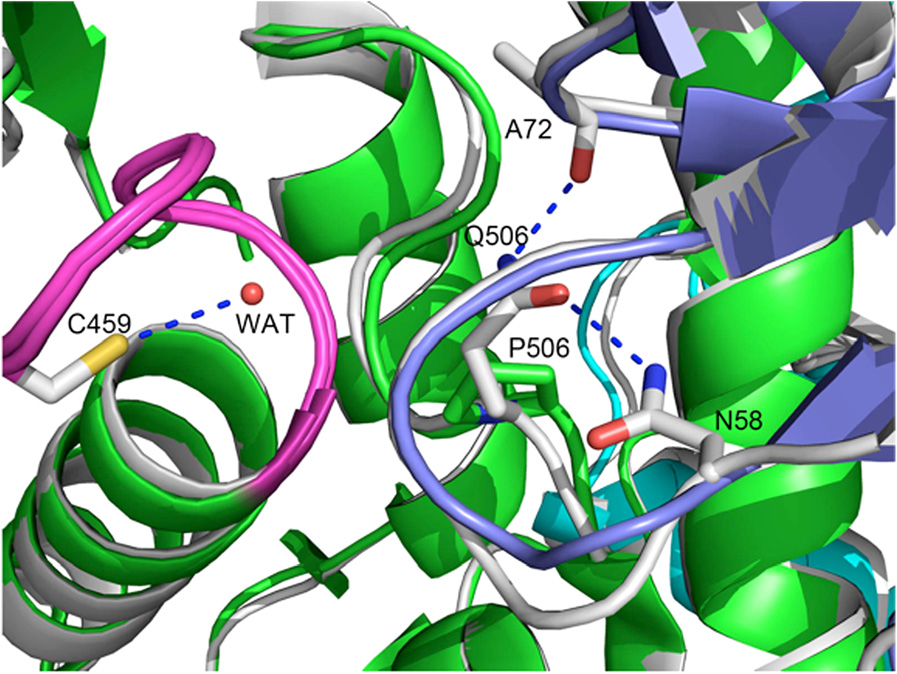
**Q506 is necessary to position and activate a water molecule for hydrolysis of the phospho-enzyme intermediate (wild type).** The Q506P mutant loses this functionality. Q506 also forms two important hydrogen bonds with Asn58 and Ala72 from the N-SH2 domain (colored in blue), which connect the N-SH2 domain to the PTP domain (colored in green). In the Q506P structure, these connections no longer are present. The P-loop (458–464) is highlighted in magenta.

Residue 506 was located in the interface between the PTP and N-SH2 domains. In the WT structure, Q506 formed two important hydrogen bonds, with the main chain of Ala72 and with the side chain of Asn58 in the N-SH2 domain. Together with Tyr279, Gln506 also plays an important role in PTP catalysis by binding the tyrosine side chain of the substrate [44] and by helping to properly position a water molecule for hydrolysis of the thiophosphate intermediate (Figure 7). In the Q506P mutant structure, the proline mutation abolished the two hydrogen bonds between the PTP and N-SH2 domain, which predicts that this mutant should be more “open” than WT SHP2. However, this mutation also disrupts the C459-H_2_O-Q506 interaction. As a result, the water molecule needed in the second step of catalysis probably cannot be positioned properly. Consistent with this notion, basal PTP activity and PTP activity in the presence of an N-SH2 domain binding pTyr peptide are slightly lower (when measured against the artificial substrate pNPP) than in WT SHP2 [29]. We also monitored the activity of the isolated catalytic domain of the Q506P mutant. In accord with our structural data, the k_cat_ of Q506P was ~15–fold lower and the K_m_ about 2× higher than in WT SHP2 (Table 2).

## Conclusion

SHP2 is regulated by a molecular switch mechanism that controls its catalytic activity. Upon binding to a tyrosine-phosphorylated binding partner for its SH2 domains, the N-terminal SH2 domain is released from the PTP domain, activating the enzyme. This elegant mechanism ensures that PTP activity is delivered to the right place in the cell at the right time. Remarkably, germ line mutations that disrupt this regulatory machinery in different ways result in distinct disease syndromes. The crystal structures of “true” WT SHP2 and five NS/LS-associated SHP2 mutants reported herein provide direct comparisons of the local conformational changes caused by each mutation. Our structural observations are in agreement with, and can provide mechanistic insight into, the previously reported catalytic properties of these mutants. For example, mutation of D61G in the N-SH2 domain significantly impacts SHP2 activity because this residue is located at the N-SH2/PTP domain interface and its alteration weakens key interactions between the two domains. On the other hand, our data suggest that the C-SH2 domain mutation E139D might interfere with SHP2 binding to tryrosine-phosphorylated ligands. The other three mutants, Y279C, N308D and Q506P, are located in PTP domain, and the local conformational changes induced by each mutation provide insight into their abnormal catalytic properties. The results of our research provide structural insights into this medically important target and could aid in future structure-based drug discovery programs.

### Availability of supporting data

The coordinates and diffraction data for SHP2 wild type and mutant crystal structures are available in Protein Data Bank (http://www.rcsb.org/pdb).

## Competing interest

The authors declare that they have no competing interests.

## Authors’ contribution

QW carried out crystal structure determination, crystallographic refinement, structure analysis, and drafted the manuscript. WX performed assay studies and participated in the drafting of the manuscript. RV and HA performed protein sample preparation and structure analysis. LA carried out crystallization studies. RM performed construct design and cloning experiments for the mutants and wild type structures. BKP performed X-ray diffraction synchrotron experiments and participated in structure analysis. EFP performed structure analysis and participated in drafting the manuscript. BGN, NYC conceived of the study, participated in its design and coordination, and drafted the manuscript. All authors read and approved the final manuscript.

## Supplementary Material

Additional file 1 **Description S1.** SHP2 cloning and mutagenesis. Click here for file

Additional file 2: Table S1 Buffers used for purification of SHP2 mutants. Click here for file

Additional file 3: Figure S1 Activities of full-length WT and mutant SHP2 studied in this manuscript (from references [29,33]). The *in vitro* catalytic activities of the indicated GST-SHP2-FLAG proteins were measured using the artificial substrate ^32^P-labeled reduced carboxamido-methylated and –maleylated lysozyme (^32^P-RCML) in the absence or presence of an insulin receptor substrate-1-derived peptide containing phospho-tyrosine-1172 (pY1172) (100 μM). The pY1172 peptide binds to the N-SH2 domain, which in turn “opens up” the enzyme. Click here for file
