# Supplementary material for: Structural insights into Noonan/LEOPARD syndrome-related mutants of protein-tyrosine phosphatase SHP2 (PTPN11)
Source: BMC Struct Biol. 2014 Mar 14;14:10. doi: 10.1186/1472-6807-14-10 (PMC4007598; doi:10.1186/1472-6807-14-10)
Supplement: Additional file 1 — Description S1. SHP2 cloning and mutagenesis. [file 1472-6807-14-10-S1.doc]

Supplementary material

Cloning

Primer combinations:

M1 Fw 5’-TACTTCCAATCCATGACATCGCGGAGATGG-3’

G539 Rev 5’- TATCCACCTTTACTGTTACCCTTTCCTCTTGCTTTT-3’

Mutation D61G

Fw D61G 5’-AACACTGGTGGTTACTATGAC-3’,

Rev D61G 5’-GTCATAGTAACCACCAGTGTT-3’

Mutation E139D

Fw E139D 5’- TTTCTTGTACGAGACAGCCAGAGCCAC-3’

Rev E139D 5’- GTGGCTCTGGCTGTCTCGTACAAGAAA-3’

Mutation Y279C

Fw Y279C 5’- AACAAAAATAGATGTAAAAACATCCTGC-3’

Rev Y279C 5’- GCAGGATGTTTTTACATCTATTTTTGTT-3’

Mutation N308D

Fw N308D 5’- ATCAATGCAGATATCATCATG-3’

Rev N308D 5’- CATGATGATATCTGCATTGAT-3’

Mutation Q506P

Fw Q506P 5’- GGGATGGTCCCCACAGAAGCA-3’

Rev Q506P 5’- TGCTTCTGTGGGGACCATCCC-3’
