# Supplementary material for: Structural insights into Noonan/LEOPARD syndrome-related mutants of protein-tyrosine phosphatase SHP2 (PTPN11)
Source: BMC Struct Biol. 2014 Mar 14;14:10. doi: 10.1186/1472-6807-14-10 (PMC4007598; doi:10.1186/1472-6807-14-10)
Supplement: Additional file 2: Table S1 — Buffers used for purification of SHP2 mutants. [file 1472-6807-14-10-S2.doc]

Table S1. Buffers used for purification of SHP2 mutants.

|  | D61G, E139D, Y279C | N308D | Q506P |
| --- | --- | --- | --- |
| Binding Buffer | 100 mM Tris-Cl, pH 8.0, 500 mM NaCl, 5% glycerol, 0.2 mM TCEP, 0.5% CHAPS | 100 mM HEPES, pH 7.5, 500 mM NaCl, 5% glycerol, 0.2 mM TCEP, 0.5% CHAPS | 100 mM Tris-Cl, pH 8.0, 500 mM NaCl, 5% glycerol, 0.2 mM TCEP, 0.5% CHAPS |
| Wash Buffer A | 100 mM Tris-Cl, pH 8.0, 500 mM NaCl, 5% glycerol, 0.2 mM TCEP, 15mM imidazole, 0.25% CHAPS | 50 mM HEPES, pH 7.5, 500 mM NaCl, 5% glycerol, 15 mM imidazole, 0.2 mM TCEP, 0.5% CHAPS | 100 mM Tris-Cl, pH 8.0, 500 mM NaCl, 5% glycerol, 0.2 mM TCEP, 3 M urea |
| Wash Buffer B | 100 mM Tris-Cl, pH 8.0, 500 mM NaCl, 5% glycerol, 0.2 mM TCEP, 15mM imidazole | 50 mM HEPES, pH 7.5, 500 mM NaCl, 5% glycerol, 0.2 mM TCEP, 15 mM imidazole | 100 mM Tris-Cl, pH 8.0, 500 mM NaCl, 5% glycerol, 0.2 mM TCEP, 15mM imidazole |
| Elution Buffer | 50 mM Tris-Cl, pH 8.0, 500 mM NaCl, 5% glycerol, 0.2 mM TCEP, 300mM imidazole | 50 mM HEPES, pH 7.5, 500 mM NaCl, 5% glycerol, 0.2 mM TCEP, 250 mM imidazole | 100 mM Tris-Cl, pH 8.0, 500 mM NaCl, 5% glycerol, 0.2 mM TCEP, 300mM imidazole |
| Gel Filtration Buffer | 10mM Tris, pH 8.0, 100 mM NaCl, 5mM DTT | 10mM Tris, pH 8.0, 100 mM NaCl, 5mM DTT | 10mM Tris, pH 8.0, 100 mM NaCl, 5mM DTT |
