# Supplementary figures and images for: Structural insights into Noonan/LEOPARD syndrome-related mutants of protein-tyrosine phosphatase SHP2 (PTPN11)
Source: BMC Struct Biol. 2014 Mar 14;14:10. doi: 10.1186/1472-6807-14-10 (PMC4007598; doi:10.1186/1472-6807-14-10)

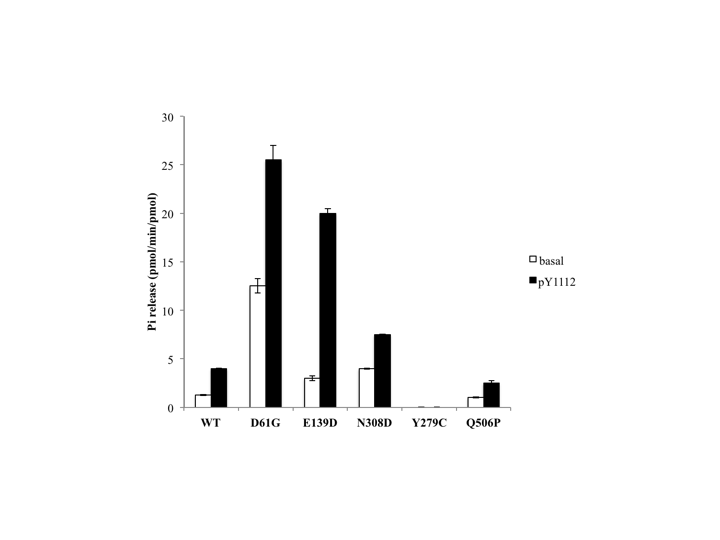

Supplement: Additional file 3: Figure S1 — Activities of full-length WT and mutant SHP2 studied in this manuscript (from references [29,33]). The in vitro catalytic activities of the indicated GST-SHP2-FLAG proteins were measured using the artificial substrate 32P-labeled reduced carboxamido-methylated and –maleylated lysozyme (32P-RCML) in the absence or presence of an insulin receptor substrate-1-derived peptide containing phospho-tyrosine-1172 (pY1172) (100 μM). The pY1172 peptide binds to the N-SH2 domain, which in turn “opens up” the enzyme. [file 1472-6807-14-10-S3.tiff]
